# Dysregulation of immune response in otitis media

**DOI:** 10.1017/erm.2021.10

**Published:** 2021-08-18

**Authors:** Michael W. Mather, Steven Powell, Benjamin Talks, Chris Ward, Colin D. Bingle, Muzlifah Haniffa, Jason Powell

**Affiliations:** 1 Faculty of Medical Sciences, Institute of Biosciences, Newcastle Medical School, Framlington Place, Newcastle-upon-Tyne, NE2 4HH, UK; 2 Department of Paediatric Otolaryngology, Great North Children’s Hospital, Queen Victoria Road, Newcastle upon Tyne, NE1 4LP, UK; 3 Faculty of Medical Sciences, Translational and Clinical Research Institute, Newcastle Medical School, Framlington Place, Newcastle-upon-Tyne, NE2 4HH, UK; 4 Department of Infection, Immunity and Cardiovascular Disease, The Medical School, The University of Sheffield, Beech Hill Road, Sheffield, S10 2RX, UK

**Keywords:** Adaptive immunity, antigen presentation, epithelium, inflammation mediators, innate immunity, otitis media, signal transduction

## Abstract

**Objective:**

Otitis media (OM) is a common reason for children to be prescribed antibiotics and undergo surgery but a thorough understanding of disease mechanisms is lacking. We evaluate the evidence of a dysregulated immune response in the pathogenesis of OM.

**Methods:**

A comprehensive systematic review of the literature using search terms [otitis media OR glue ear OR AOM OR OME] OR [middle ear AND (infection OR inflammation)] which were run through Medline and Embase via Ovid, including both human and animal studies. In total, 82 955 studies underwent automated filtering followed by manual screening. One hundred studies were included in the review.

**Results:**

Most studies were based on in vitro or animal work. Abnormalities in pathogen detection pathways, such as Toll-like receptors, have confirmed roles in OM. The aetiology of OM, its chronic subgroups (chronic OM, persistent OM with effusion) and recurrent acute OM is complex; however, inflammatory signalling mechanisms are frequently implicated. Host epithelium likely plays a crucial role, but the characterisation of human middle ear tissue lags behind that of other anatomical subsites.

**Conclusions:**

Translational research for OM presently falls far behind its clinical importance. This has likely hindered the development of new diagnostic and treatment modalities. Further work is urgently required; particularly to disentangle the respective immune pathologies in the clinically observed phenotypes and thereby work towards more personalised treatments.

## Introduction

Otitis media (OM) is an umbrella term for inflammation of the middle ear and is a leading cause of medical consultations, antibiotic prescriptions and surgery in children (Ref. 1). Worldwide there are in excess of 700 million cases of acute OM per year, with the majority occurring in children under 5 years old. Most children will be affected by OM at some point; over 60% of children will have experienced at least one episode of acute OM by their first birthday, and more than 15% will have suffered recurrent acute episodes (Ref. 2).

Most patients exhibit complete resolution of infective symptoms of acute OM within a few days. However, a subgroup subsequently suffers from ongoing pathology, including (1) recurrent acute OM, in which there are regular episodes of acute infection causing pain but with resolution of symptoms in between. (2) A persistent fluid effusion in the middle ear (OM with effusion; OME); this impairs the conduction of sound through the middle ear which leads to hearing loss and is associated with a negative impact on language acquisition, behaviour and progress at school (Ref. 3). (3) Chronic OM (COM); of which there are two subtypes – mucosal and squamous. Mucosal COM indicates an inflamed middle ear with an associated tympanic membrane (TM) perforation. Mucosal COM can be either active (producing discharge) or inactive (dry). Squamous COM is caused by severe retraction of the TM and is associated with a buildup of keratin (known as cholesteatoma) which can be locally erosive. These terms are summarised in the attached glossary (Box 1). These subgroups are not exclusive and may overlap and change with time, or indeed lead to one another.

Despite the considerable burden of disease, it presently remains unclear why the majority of children make a spontaneous recovery from limited episodes of acute OM but a subset, who are frequently otherwise apparently immunocompetent, develop recurrent acute or chronic disease.

In health, the human immune system regulates the response to infectious stimuli by providing robust action against non-self-antigens whilst exerting tolerance to the host’s own cells. However, at many anatomical sites, dysfunction of this process drives many inflammatory disease processes (Ref. 4). Here we review the evidence of this dysregulation in the transition from sporadic uncomplicated acute OM to recurrent acute or chronic forms of OM. We suggest that understanding this process will be key to developing novel therapeutics, which have remained largely unchanged for over 50 years.

## Methods

A comprehensive systematic review of the literature using search terms [otitis media OR glue ear OR AOM OR OME] OR [middle ear AND (infection OR inflammation)] were run through Medline and Embase via Ovid. Both human and animal studies were included. In total, 82 955 studies underwent automated filtering for the English language only articles, full-text availability only, dated 1990–2021. Following de-duplication, 2557 abstracts were manually screened. One hundred studies were included in the review, including those identified in bibliographies of returned studies for a comprehensive literature review. A summary PRISMA diagram is available in Supplementary materials.

### Pathogen detection in the middle ear

#### Acute and recurrent acute otitis media

An initial episode of acute OM may be viral or bacterial in nature, and the former can predispose to the latter (Ref. 5). Clinically it is not immediately apparent which is the causative pathogen in each case; however, a bacterial species is identified in approximately two-thirds of sampled middle ears in the acute setting (Ref. 6). The most common bacterial otopathogens in acute OM are shown in Table 1.

**Fig. 1:**
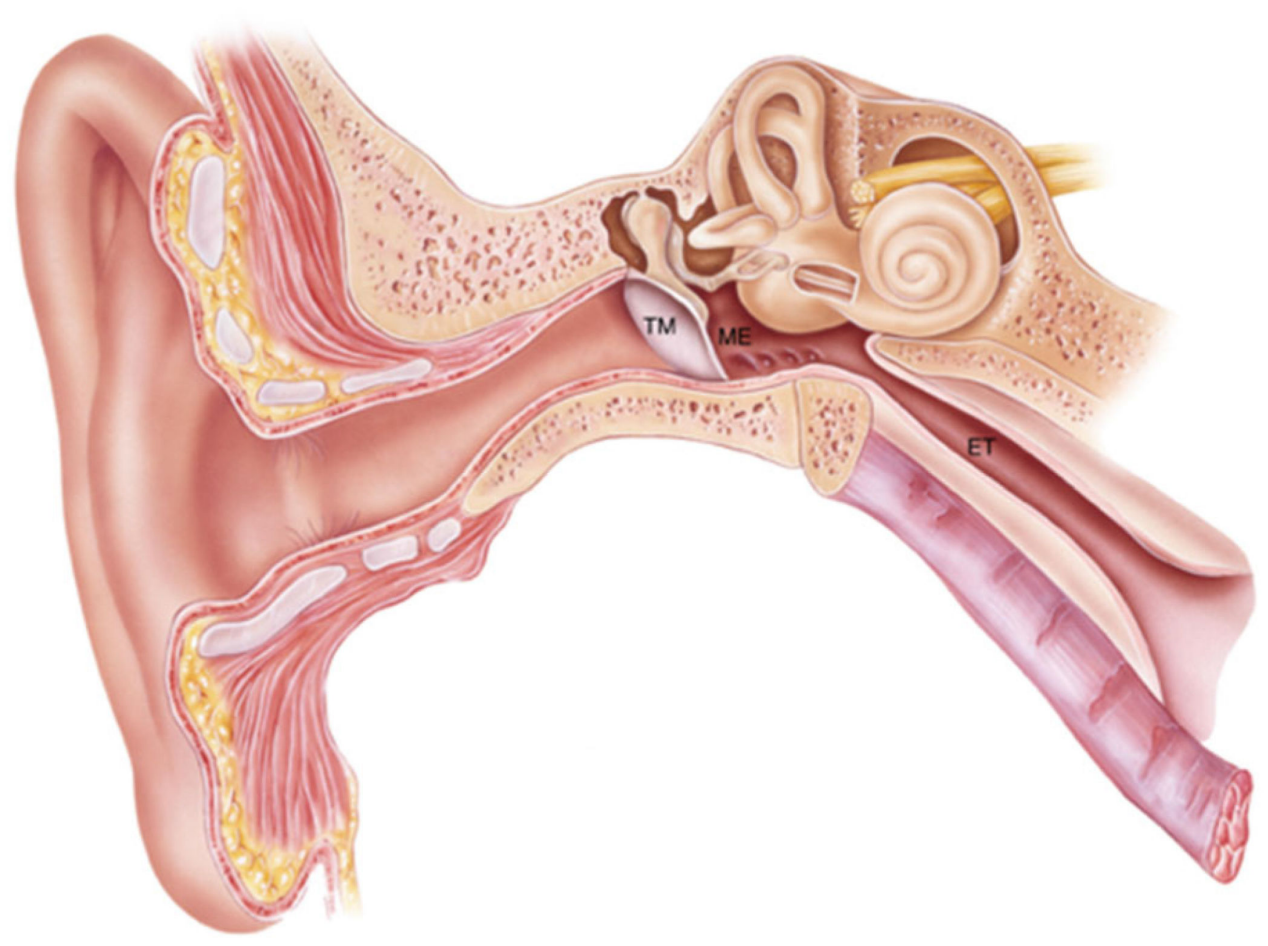
The human ear. OM occurs within the middle ear (ME) cavity; the space behind the tympanic membrane (TM) which communicates with the post-nasal space via the Eustachian tube (ET).

The origin of these organisms is likely from the nasopharynx via the Eustachian tube (Fig. 1), as demonstrated by studies which show no significant difference in microbiome diversity between nasal washings and middle ear effusion during an episode of acute OM (Ref. 7). One theory is that initial viral infection of the nasopharynx leads to an inflammatory reaction in the Eustachian tube, causing Eustachian tube dysfunction, and translocation of bacteria into the middle ear cavity (Refs 8, 9). This is also consistent with the notion that the Eustachian tube is shorter and more horizontal in children than adults, which could facilitate bacterial translocation and contribute to their relatively higher risk of OM.

In order to generate an immune response, the host must first detect the presenting pathogen. This is mediated by patternrecognition receptors (PRRs) such as membrane-bound Toll-like receptors (TLRs) or C-type lectins which are expressed on diverse host cell types including epithelial cells, as well as classical immune sentinels such as macrophages and dendritic cells. They detect invariant microbial motifs or pathogen-associated molecular patterns (PAMPs) in the extracellular space and trigger signal transduction via adaptor proteins such as myeloid differentiation primary response protein 88 (MyD88), leading to a pro-inflammatory response (Ref. 10).

**Fig. 2:**
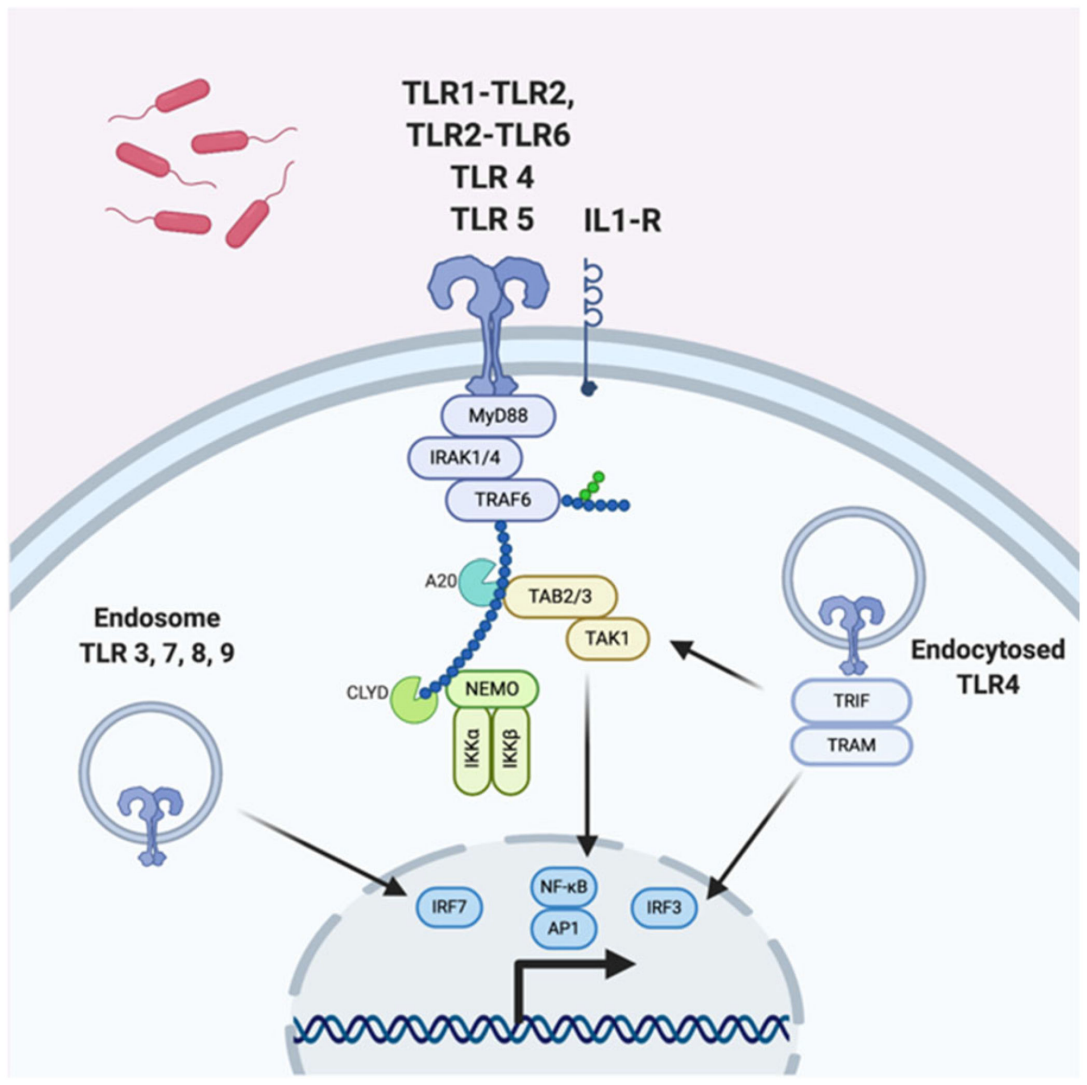
TLR signalling mechanisms; mouse and human. Extracellular pathogens are detected by one of a range of TLRs which trigger downstream signalling via adaptor proteins including MyD88 and IRAK1/4. This leads to the upregulation of genes involved in generating a pro-inflammatory response. Adapted from O’Neill *et al*. (Ref. 10).

Multiple defects in TLR signalling (Fig. 2) have been described in animal models of OM. *Tlr9* knockout models show a prolonged inflammatory response in the middle ear and delayed bacterial clearance (Ref. 11), and *Tlr4* knockout models have reduced capacity for TNF production, a late increase in *IL-10* expression, and failure to eradicate bacteria (Ref. 12). Similarly, *Tlr2* knockout models demonstrate a more severe OM phenotype compared with wildtypes – including a higher potential for systemic sepsis. They also have reduced macrophage recruitment and produce fewer pro-inflammatory mediators, which is hypothesised to lead to impaired bacterial clearance (Refs 13, 14, 15). Whilst the majority of studies on TLR signalling in OM have been conducted in mice, polymorphisms in *TLR4* have also been observed clinically in children with a propensity towards recurrent acute OM in population-based studies (Ref. 16).

Functional signal transduction proteins downstream from TLRs, such as myeloid differentiation primary response gene 88 (MyD88) and Tir-domain-containing adaptor inducing interferon *β* (TRIF), have been shown to be essential for resolution of OM in mice (Refs 17, 18). Similarly, patients with mutations in *MyD88* and *IRAK4*, which is also part of TLR-mediated signal transduction, have a tendency towards recurrent AOM (Ref. 19). The multiple positive findings in this pathway together suggest defective TLR-mediated pathogen detection and subsequent signal transduction impairs the host response to infection in the middle ear cleft.

However, even in situations of fully functional TLRs, it has been identified that otopathogens themselves have mechanisms to overcome TLR-mediated detection; for example, *Moraxella catarrhalis* has been shown to deliver outer membrane vesicles in response to TLR2 binding, and surface proteins – such as ubiquitous surface protein A1 (UspA1) – modulate the inflammatory response of epithelial cells. It therefore seems that otopathogens can interact with specific TLRs to subdue the host response to infection once they have infiltrated the tissue, making them challenging to eradicate (Ref. 20).

#### Otitis media with effusion

Most research efforts to date have focused on pathogen detection in recurrent AOM and defects in these pathways are much less clearly described in OME. Indeed, it remains unclear whether OME is initiated by acute infection and then maintained by non-infective host factors, or whether failure to fully eradicate an infective nidus leads to persistent inflammation.

In favour of the former, there are mechanisms which can lead to persistent ‘sterile’ inflammation in the middle ear. Debris from damaged cells, for example, remnants of bacterial cell walls (Refs 21, 22), can trigger an inflammatory response via so-called danger-associated molecular patterns (DAMPs), which may contribute to COM subtypes, in the absence of clinical evidence of active infection.

In favour of the persistent infective stimulation theory, bacterial biofilms have been detected in both the middle ear (Ref. 23) and on adenoids (Ref. 24) (nasopharyngeal lymphoid tissue closely associated with the Eustachian tube orifice) of children with chronic middle ear disease more frequently than those without. These biofilms are extracellular matrices which house bacterial colonies and confer antimicrobial resistance (Ref. 25). It is plausible that such a system enables bacteria to escape host immune-mediated destruction and lead to persistent inflammation. However, even in effusions which do not have evidence of biofilm formation, live bacteria are frequently found (Ref. 26).

Whichever the stimulus, both DAMP and PAMP signalling pathways converge on the activation of common pro-inflammatory pathways, such as NF-κB and activator protein-1 (AP-1) or interferon regulatory factor 3 (IRF3) which act as transcription factors to initiate a pro-inflammatory and type 1 interferon response which are essential for effective bacterial and viral clearance (Ref. 27).

#### Chronic otitis media

Eustachian tube opening is recognised as important for appropriate ventilation of the middle ear and dysfunction of this process has been implicated in chronic phenotypes of OM (Ref. 28) – possibly via increasing the risk of pathogen translocation into the middle ear. Further, it has been shown that intracellular pathogen recognition proteins have a demonstrated role in murine OM; for example, nucleotide-binding oligomerisation domain-like (NOD) receptors. Both NOD1 and NOD2-deficient mice show prolonged OM; specifically, NOD1-deficient mice demonstrate reduced macrophage recruitment and delayed inflammatory responses from neutrophils, and NOD2-deficient mice show reduced overall leukocyte influx and impaired bacterial clearance (Ref. 29). Evidence of NOD deficiency in humans causing OM is lacking but interestingly human samples of cholesteatoma, which is a sequela of chronic OM in which there is an abnormal accumulation of keratinizing squamous epithelium in the middle ear, show the upregulation of NOD2 (Ref. 30) – suggesting the role of PRRs in progression from OM to chronic complications may change over the course of disease.

Furthermore, the bacteria identified in COM and OME (Table 2) frequently differ from those found in AOM. Therefore, it seems likely that a complex interplay of both host immunity and extrinsic microbiological factors is involved in the development of COM, but this remains an area in need of further work.

### Middle ear inflammation

#### Acute and recurrent acute otitis media

The host inflammatory response involves a diverse repertoire of effector cells and cytokines. Neutrophils, the most abundant immune effector cells in humans, are the primary cell type initially recruited to sites of infection. They are attracted by chemotactic stimuli, such as CXCL8 (IL-8), which is secreted by tissue resident macrophages or indeed infected tissue itself. Consequently, IL-8 in middle ear effusion is significantly more elevated in culture-positive acute OM than culture-negative OM (Ref. 32), and is higher in the effusion of acute OM-prone children than those who are not acute OM-prone (Refs 33, 34). IL-8 is also consistently higher in acute OM and COM than OME, and is associated with higher neutrophil counts in middle ear effusion (Ref. 35). Therefore, a means to modulate IL-8 signalling may be beneficial and would be a valuable area of further research – particularly in the context of those who are otitis prone.

Epithelial cells have important interactions with immune cells and consequently modulate airway inflammation. For example, healthy airway epithelium is a potent inhibitor of dendritic cell-induced T-cell activation (Ref. 36). Furthermore, epithelial cell damage, such as by viral infection, leads to loss of this T-cell inhibition and consequent airway inflammation (Refs 37, 38). It logically proceeds that epithelial damage in the middle ear cavity – for example, an episode of viral or bacterial acute OM – could impair T-cell regulation and facilitate tissue inflammation. It may also be that an initial viral infection leads to ciliopathy which predisposes to secondary bacterial infection as occurs elsewhere in the respiratory tree (Ref. 39).

Insight from other upper airway mucosal sites may also be informative in understanding epithelial inflammation in OM. In chronic rhinosinusitis, for example, there is now emerging evidence of distinct endotypes which are each mediated by particular innate lymphoid cell (ILC1/2/3) and T helper cell (Th1/Th2/ Th17) subgroups. It logically follows that treatments for these subgroups may differ – particularly with the advent of targeted monoclonal antibody or ‘biologic’ therapies (Ref. 40). Analogous molecular subgrouping in OM is lacking, but similar mechanisms seem plausible and may offer new routes to more personalised treatments.

Whilst they do not yet inform OM subgroups, the differential function of T-cell subgroups in OM has already been studied systemically; even from birth, enhanced T helper 1 and 2 cytokine production has been observed to be associated with a subsequently lower risk of middle ear infections in infancy (Ref. 41). Conversely, T cells from adenoids of children with recurrent acute OM appear to be more readily stimulated by bacteria and are more productive of IFN-γ than those from children without OM (Refs 42, 43). Therefore, there appears to be a dichotomy between whether elevated T-cell activity is protective against, or is a risk factor for, recurrent acute OM. It may be that the relevant abundances of specific subgroups of T cells are important. Indeed, there are suggestions that deficits in T-cell memory specifically contribute (Ref. 44). It may also be that there are divergences between systemic and tissue-resident T cells, and this would be an interesting avenue for future research.

The complement system, a protein constituent of serum and tissue fluid responsible for opsonisation and killing of bacteria, is also involved in governing the recruitment of immune effector cells in the middle ear. Knockout studies in mice have demonstrated increased susceptibility to *Streptococcus pneumoniae-induced* OM with impairment of either the classical or alternative complement pathways (Ref. 45). Indeed, S. *pneumoniae* strains able to restrict complement binding were more likely to cause invasive disease in a chinchilla model of OM (Ref. 46). Polymorphisms in mannose-binding lectin, which is responsible for complement-mediated pathogen surface recognition, have been identified as risk factors for recurrent acute OM; however, these seem to be mainly in younger age children (i.e. <2 years) when maternally-derived antibodies are no longer effective, but when adaptive immunity is not yet mature (Refs 47, 48, 49).

In humans, much of the localised inflammatory signalling triggered by immune infiltration is because of the production of cytokines. Although viruses are known to cause acute OM, their capacity to induce such pro-inflammatory mediators detectable in middle ear effusion appears to be limited compared with bacterial otopathogens (Ref. 50). In acute bacterial OM, there is vigorous production of proinflammatory cytokines including IL1*β*, TNF and IL8, which rapidly decrease once bacteria are fragmented (Ref. 51). Defective production of such cytokines appears to confer an acute otitis-prone phenotype; particularly with polymorphisms in *TNFA, IL6* and *IL10* (Refs 52, 53). This suggests that a robust proinflammatory response is essential for bacterial clearance and defects in this process enable recurrent acute infections. This is supported by murine knock-out models affecting TNF signalling pathways, for example, *Tgif*^−/−^ (Ref. 54), *Fbxo11*^−/−^ (Ref. 55) and *Evi1*^−/−^ (Ref. 56) which all demonstrate increased OM susceptibility. Interestingly, whilst TNF-deficient mice have defects in downstream protein production, for example, CCL3, the addition of recombinant TNF only partially restores macrophage phagocytic capacity but recombinant CCL3 completely reverses the impairment (Ref. 57). This is consistent with further work that shows *Ccl3*^−/−^ mice with induced OM have compromised bacterial clearance and thickening of middle ear mucosa, along with infection by atypical otopathogens, such as *Klebsiella pneumoniae*, increased bacterial load in the nasopharynx, and disrupted nasopharyngeal microbiome (Refs 58, 59). This suggests that whilst TNF has pleiotropic effects on innate immune response, targeting particular downstream mediators may be more useful for therapeutic manipulation.

#### Otitis media with effusion

It remains uncertain whether OME is invariably a sequela of AOM which fails to fully resolve, or whether there are distinct instigator mechanisms at the beginning of episodes which subsequently manifest an OME phenotype. However, it is clear that complex multi-factorial defects in host immunity play a role in the development of OME. For example, macrophages extracted from chronic effusions have been shown to suppress the activities of lymphocytes; making them permissive to chronic infection (Ref. 60). Additionally, middle ear mucosal samples from children with chronic effusion have demonstrated intense complement activation (Ref. 61). It has also been observed that there are higher serum levels of some interleukins (e.g. IL-5) in children with OME than controls (Ref. 62) but, perhaps surprisingly, differences in serum IgG levels do not seem to play a role (Ref. 47). There are suggestions that the development of chronic mucosal disease might be a function of duration of inflammation, rather than the intensity of cytokine production (Ref. 63). Indeed, duration of inflammatory signalling may be relatively prolonged in the context of dense nasopharyngeal colonisation; which is in keeping with known risk factors for OME (Ref. 64).

The mucosal surfaces of the upper respiratory tract and middle ear themselves have evolved multiple ways to protect the host from infection and one key mechanism is the production of mucins. These large glycoproteins provide a key barrier function and are responsive to a range of inflammatory cytokines (Ref. 65). It has been observed that the presence of otopathogens triggers the upregulation of gel-forming mucins and indeed that polymicrobial infection can act synergistically to increase this further (Ref. 66). This upregulation of mucin genes in the middle ear, such as *MUC2, MUC5AC* and *MUC5B*, contributes to effusion viscosity (Ref. 67). MUC5B, in particular, seems to be especially high in mucoid as compared with serous effusions (Refs 68, 69) Interestingly, *MUC5B*^−/−^ mice show chronic middle ear infection and persistent inflammation (Ref. 70). So, whilst overproduction of some mucins is associated with viscous effusion formation which impairs the transmission of sounds through the middle ear and results in a conductive hearing loss, others likely play a critical role in capturing and removing pathogens. Indeed, at other upper airway sites, it has been identified that mucins may also lead to immune cell dysfunction and bacterial growth (Ref. 71).

Another key mechanism of mucosal defence is the barrier and biomechanical function of epithelial cells. The middle ear epithelium, like that of the lower airways, has a tight network of intercellular apical junctional complexes which regulate the paracellular secretion of antimicrobial peptides, in addition to preventing the ingress of undesired pathogens, allergens and particulate matter in order to avoid inappropriate tissue inflammation (Ref. 72). As OM has clinical correlations with infection, allergy and environmental contamination (Ref. 1), it seems likely that the barrier function of middle ear epithelium is likely critical in regulating tissue homeostasis.

However, it remains to be fully elucidated how the fluid effusions are actually generated in OME. Some studies have suggested that complement anaphylatoxins (such as C3a and C5a), which are responsible for chemoattraction of inflammatory cells, are significantly more elevated in middle ear effusion in persistent OM but whether this is cause or effect remains unclear (Ref. 73). Other work has identified that protein derivatives from neutrophils known as neutrophil extracellular traps (NETs), widely described as an innate defence mechanism in pulmonary infections, surprisingly appear to facilitate secondary pneumococcal infection in the middle ear (Ref. 74). They have also been identified in middle ear effusion and appear to co-localise with MUC5B (Ref. 75) – an observation consistent with reports that NETs promote and sustain epithelial mucin secretion (Ref. 76), suggesting that NETosis may contribute to fluid viscosity in OME, even once the acute infection has resolved. Disruption of NETs may therefore be a valuable direction for resolving viscous middle ear effusions.

Alternative hypotheses can be derived from the observation that OME is more prevalent in children with concomitant allergic rhinitis than those without (Refs 77, 78), suggesting that congestion of the nasal mucosa leads to Eustachian tube dysfunction and negative middle ear pressure with consequent transudation of fluid. Allergy is also associated with an excessive Th2 response, driving high IL4 production – which is inhibitory to a Th1 (i.e. antibacterial) response, thereby permitting chronic infection and inflammation. Other observations in OME include the upregulation of hypoxia-related genes leading to increased angiogenesis (Ref. 79) and enzyme-mediated tissue damage (Ref. 80), but the relationship of these to the immune mechanisms above remain unclear.

#### Chronic otitis media

As with pathogen detection, the mechanisms governing mucosal inflammation in COM are less well understood than in AOM and OME. It is clear that the host middle ear epithelium is crucial for tissue homeostasis, the regulation of inflammation and antimicrobial defence, and is therefore likely of importance (Ref. 81). It is, however, notable that the middle ear epithelium is not homogenous in structure, and is therefore unlikely homogeneous in function. Recent work has described how mucosa morphology differs throughout the middle ear cavity from pseudostratified ciliated epithelium, with its functional capacity for bacterial eradication via mucociliary clearance around the Eustachian tube orifice, through to progressively thinner gas exchanging epithelium located more superiorly (Ref. 82). Transformation of this epithelium through partial epithelial to mesenchymal transition (EMT), that is, loss of cell-cell adhesion and apico-basal polarity in favour of mesenchymal traits of migration, invasion and producing extracellular matrix components, has been noted in samples from human cholesteatoma (Ref. 83). EMT has a clear role in the development of chronic lower airways diseases, such as chronic obstructive pulmonary disease and pulmonary fibrosis (Ref. 84); so it is plausible that such a mechanism is at work in chronic middle ear inflammation. This is also consistent with observations that known risk factors for OM, such as smoking, are known to drive EMT (Ref. 85). However, as not all classical features of EMT are observed in cholesteatoma samples (e.g. tight junction formation and terminal differentiation are not affected), it may be that this represents a distinct pathological process compared with that observed in the other EMT-related biological processes, such as normal development, cancers and chronic lung disease (Ref. 86).

Persistent infection/inflammation can lead to tissue remodelling in the middle ear; for example, mucous glands become more prevalent in COM (Ref. 87). Paradoxically, over time, these mucous glands can degenerate; diminishing the capacity for mucin production, which may have important antimicrobial properties (Ref. 88). Indeed, it has been shown that the stroma in COM is infiltrated with macrophages and there is significantly lower expression of *Tlr2, 4* and 5, but the upregulation of *TNF-α, IL-1β, IFN-γ* and *IL-6* compared with healthy ears. Together these indicate a pro-inflammatory milieu which, over time, leads to histological and functional changes which diminish the host’s capacity to respond to infection in those with COM (Ref. 89). There remains a question about whether these observations are driven by a dysfunctional host immune response or whether persistent infection is directly causing these changes. This would be a valuable area for future research.

### Anti-inflammatory resolution in the middle ear

The dampening of inflammation in disease resolution has known importance at many anatomical subsites but an understanding of this in the middle ear is presently lacking. Generalised mechanisms include the production of anti-inflammatory mediators, abolition of neutrophil recruitment, neutrophil apoptosis, chemokine depletion and alternative activation of macrophages (Ref. 90). It seems likely that similar mechanisms are at work in the healthy middle ear, but experimental confirmation is awaited. It is known that anti-inflammatory cytokine genes, such as *IL10*, are upregulated on induction of acute OM, particularly with episodes secondary to S. *pneumoniae* (Ref. 91). Specific analogues of such pathways in AOM remain to be trialled and inhibitors of pro-inflammatory cytokines, including TNF-α, have only been trialled in animal models (Ref. 92).

Multiple medical treatments to resolve chronic inflammation in OME have been tested but the translational potential to date has been limited. For example, whilst steroids showed promise in reducing inflammation in a murine model of OM (Ref. 93), the OSTRICH trial; a double-blinded randomised trial comparing a short course of oral steroids with placebo in children with OME, did not demonstrate significant clinical benefit (Ref. 94). However, one in 14 children did have an improvement in hearing thresholds, suggesting a possible useful mechanistic element to suppression of inflammation in the middle ear.

Other therapeutic agents aimed at reducing middle ear inflammation have been investigated in animal models and cell lines. For example, Polymyxin B which binds to and inactivates bacterial endotoxin (found on the surface of Gram-negative bacteria) has shown promise in reducing cellular infiltrate, effusion volume and mucosal oedema in a guinea pig model of OME (Ref. 95), as has the manipulation of pathways which regulate mucosal inflammation, such as stimulation of the peroxisome proliferator-activated receptor-gamma (PPAR*γ*) pathway with agonist Ciglitazone which dampens the inflammatory response in the human middle ear epithelial cell (HMEECs) line in vitro (Ref. 96). Similarly, blockade of granulocyte/macrophage colony-stimulating factor – a potent proinflammatory peptide – results in reduced infiltration of inflammatory cells in middle ear mucosa in a murine model of OM (Ref. 97). Metformin, most commonly used in the treatment of diabetes mellitus, has also been tested in vitro because of the reports of anti-inflammatory effects. Indeed in HMEECs treated with LPS, metformin suppressed the production of TNF-α, COX-2 and intracellular reactive oxygen species (Ref. 98). Another agent, vinpocetine, which suppresses inflammation induced by *S. pneumoniae* through inhibition of ERK (Ref. 99) reduces middle ear inflammation in a murine model of OM. To date, none of these have been trialled in human OM.

**Fig. 3:**
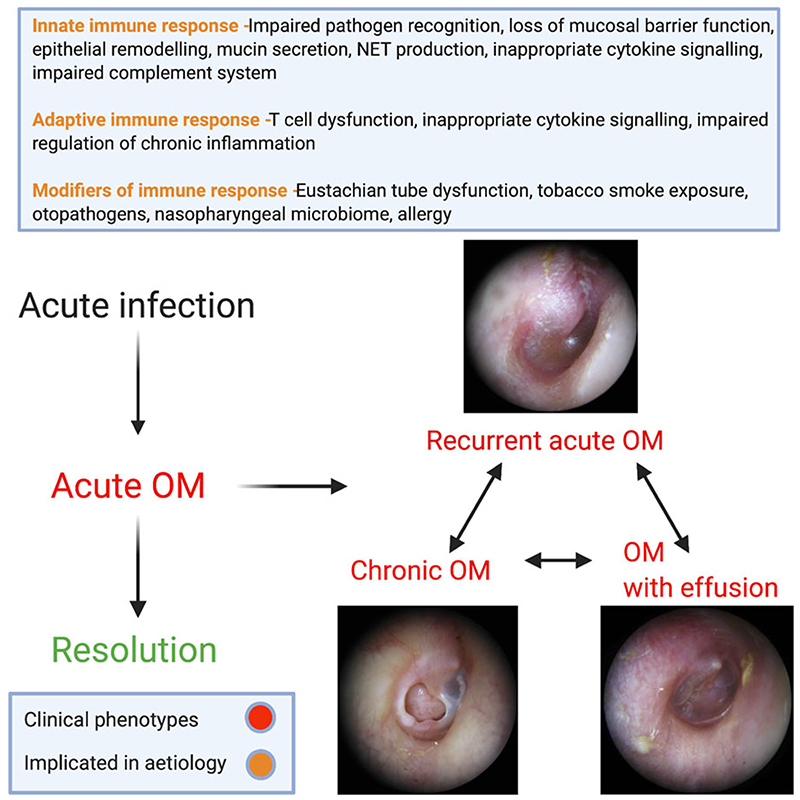
Summary of implicated mechanisms of disease in subgroups of OM.

A summary of the discussed mechanism of disease in OM subgroups is demonstrated in Figure 3.

## Conclusion and future work

OM comprises a broad spectrum of interrelated pathologies. Defects in host immunity appear to play a key role in the response to initial infection and a complex range of modifying factors determine if, and which, of the subgroups subsequently emerges. At present, the limited translational literature does not reflect the substantial clinical burden of OM. Much information to date has been derived from cell lines and animal models of OM, which may not fully reflect the human pathology. Difficulties in the acquisition of human middle ear tissue have likely played a part in this and the development of physiological systems to study OM is required. If defects in mechanisms of disease could be identified, pharmacological manipulation would be an appealing alternative to existing surgical treatments; reducing both risk and cost to patients who, until now, find themselves with limited treatment options.

## Supplementary Material

The supplementary material for this article can be found at https://doi.org/10.1017/erm.2021.10

Supplementary Material

## Figures and Tables

**Table 1:** Typical bacterial species in acute otitis media; adapted from Mather *et al.* (Ref. 6)

Bacterial species	Prevalence
*Streptococcus pneumoniae*	0.30 (CI 0.27–0.32)
*Haemophilus influenzae*	0.23 (CI 0.20–0.26)
*Moraxella catarrhalis*	0.05 (CI 0.04–0.06)

**Table 2:** Bacterial species identified in COM and OME ranked in order of prevalence; adapted from (Ref. 31) and (Ref. 26)

COM	OME
*Pseudomonas aeruginosa*	*Coagulase negative staphylococci*
*Staphylococcus aureus*	*Veillonlla spp*.
*Klebsiella pneumoniae*	*Staphylococcus aureus*
*Proteus mirabilis*	*Streptococcus pneumoniae*
*Proteus vulgaris*	*Bacillus spp*.
*Escherichia coli*	*Moraxella catarrhalis*
*Streptotoccus pneumoniae*	*Pseudomonas spp*.

## Box 1 Glossary of termsThe following terms are based upon definitions provided in (Ref. 1) and (Ref. 100).

**Table:**

Otitis media (OM)	An umbrella term for inflammation of the middle ear.
Acute otitis media (AOM)	Rapid onset signs and symptoms of middle ear inflammation.
Recurrent acute otitis media (rAOM)	≥3 discrete episodes of AOM in a 6-month period OR ≥4 discrete episodes of AOM over 12 months with at least one episode in the last 6 months
Otitis media with effusion (OME)	Fluid within the middle ear but no signs or symptoms of AOM
Glue ear	Analogous term to otitis media with effusion (OME).
Chronic otitis media (COM)	Middle ear inflammation with persistent or recurrent ear discharge. Can be subdivided into mucosal and squamous subtypes. Mucosal COM has inflamed middle ear mucosa associated with a perforated tympanic membrane (TM) and can be active (producing discharge) or inactive (dry). Squamous COM occurs due to retraction of the TM and is associated with cholesteatoma formation.
Cholesteatoma	Abnormal accumulation of keratin in the middle ear, which can be locally destructive.
Chronic suppurative otitis media (CSOM)	Analogous term to active mucosal COM.
