# Supplementary material for: Dysregulation of immune response in otitis media
Source: Expert Rev Mol Med. 2021 Aug 18;23:e10. doi: 10.1017/erm.2021.10 (PMC7612930; doi:10.1017/erm.2021.10)
Supplement: Supplementary file 1 [file S1462399421000107sup001.docx]

Supplementary materials:

Records identified from:

Databases (n = 2; Medline and EMBASE via OVID)

Registers (n = 0)

**Identification**

Records removed by automated screening (n = 80,398):

- Not English language (n=14,002)
- Full text not available (n=65,009*)*
- Not 1980-2021 (n=84)
- Duplicate articles (n=1,303)

Records identified

(n = 82,955)

**Screening**

Reports manually screened for relevance to immunology of OM

(n = 2,557)

Studies included in review

n = 100 (including additional papers from searching paper bibliographies)

**Included**

*From:*  Page MJ, McKenzie JE, Bossuyt PM, Boutron I, Hoffmann TC, Mulrow CD, et al. The PRISMA 2020 statement: an updated guideline for reporting systematic reviews. BMJ 2021;372:n71. doi: 10.1136/bmj.n71
